# Evaluating the Usage of Janus Kinase Inhibitors in Rheumatology and Its Impact on Cardiovascular Risk

**DOI:** 10.7759/cureus.65591

**Published:** 2024-07-28

**Authors:** Knkush Hakobyan, Talar Acob, Mesrop Aleksanyan, Tigran Kakhktsyan, Omar Jumaah, Sajina Prabhakaran

**Affiliations:** 1 Internal Medicine, Capital Health Regional Medical Center, Trenton, USA; 2 Oncology, Yerevan State Medical University, Yerevan, ARM; 3 Rheumatology, Capital Health Regional Medical Center, Trenton, USA

**Keywords:** lipid worsening, tofacitinib, psoriatic arthritis, rheumatoid arthritis, jak 2 kinase inhibitor

## Abstract

Background/purpose

Janus kinase (JAK) inhibitors have been widely used in treating rheumatological conditions like rheumatoid arthritis (RA) and psoriatic arthritis (PsA). Despite their efficacy, there are concerns regarding major adverse cardiovascular events (MACE) and venous thromboembolism (VTE) associated with JAK inhibitors. This study aimed to evaluate the risk of MACE, VTE, and the impact on lipid profiles in patients being treated with JAK inhibitors.

Methods

We retrospectively reviewed electronic medical records of patients aged 45-65 years old treated with Tofacitinib, Baricitinib, or Upadacitinib in a rheumatology clinic. We collected data on demographics, comorbidities, medication use, laboratory results, and cardiac complications potentially related to JAK inhibitors.

Results

Among 100 patients prescribed JAK inhibitors, 71 were included in the study (with an average treatment duration of 2.5 years). The majority of patients were white (72%), followed by Hispanic (6%), Indian (11%), African American (10%), and Asian (1%). Patients were being treated primarily for RA (57%), followed by PsA (17%), colitis (20%), and alopecia areata (6%). There were no significant cases of VTE reported, with one patient developing a pulmonary embolism (PE) during treatment while also having COVID-19, making it difficult to attribute it solely to the medication. Similarly, only one case of atrial fibrillation occurred.

However, 43% (31 patients) experienced worsening of their lipid profile, with increased cholesterol (18%), LDL (12.5%), both LDL and cholesterol (11%) or triglycerides (1.5%). In relation to diabetes mellitus (DM), 24 patients who experienced worsening of their lipid panel did not have a history of DM.

Conclusion

The study findings suggest that patients on Tofacitinib, Baricitinib, and Upadacitinib did not exhibit a high risk for MACE or DVT. However, there was a notable incidence of lipid panel worsening among patients, where 24 patients out of 31 did not have diabetes. Further research and monitoring may be needed to better understand the long-term effects of JAK inhibitors on cardiovascular health and lipid profiles in these patient populations. This real-world data reflects the current evidence that JAK inhibitors do not significantly raise the risk of MACE in patients with RA but do increase cholesterol levels in these patients that should be monitored closely.

## Introduction

Rheumatologic conditions, including rheumatoid arthritis and psoriatic arthritis, are characterized by chronic inflammation that can lead to significant morbidity and mortality. Janus kinase (JAK) inhibitors have emerged as a novel therapeutic option for managing these conditions by modulating inflammatory pathways. Despite their efficacy in controlling disease activity, concerns about their long-term safety profile have been raised, particularly regarding cardiovascular health, major adverse cardiovascular events (MACE), venous thromboembolism (VTE), and impacts on lipid profiles.

The JAK family plays a critical role in the signaling pathways of various cytokines and growth factors involved in hematopoiesis, immune function, and inflammation. JAK inhibitors specifically target the JAK enzymes implicated in the pathogenesis of several autoimmune diseases [1]. While these inhibitors offer a promising approach to disease management, their impact on the cardiovascular system remains a subject of ongoing research and debate [2].

This retrospective study aims to evaluate the usage patterns of JAK inhibitors in patients with rheumatologic conditions and to assess their impact on cardiovascular health. By analyzing clinical data, we seek to understand the correlation between JAK inhibitor therapy and cardiovascular outcomes such as myocardial infarction, stroke, VTE, and changes in lipid profiles [3].

Previous studies have suggested a potential link between JAK inhibitor therapy and increased cardiovascular risk, necessitating a comprehensive examination of patient data to elucidate these relationships further. Understanding these associations is crucial for optimizing treatment strategies and ensuring patient safety [4].

In this study, we reviewed patient records from our rheumatology clinics, focusing on those prescribed JAK inhibitors such as Tofacitinib, Baricitinib, and Upadacitinib. We analyzed demographic data, disease characteristics, treatment regimens, and cardiovascular outcomes. Our goal is to provide a detailed assessment of the cardiovascular risks associated with JAK inhibitors, contributing to the body of evidence needed to guide clinical decision-making in rheumatology.

## Materials and methods

Study design and setting

This study was a retrospective review conducted at Capital Health, Trenton, NJ. The research was carried out in the rheumatology department and electronic medical records were reviewed from the Athena medical record system. Data collection and storage were performed using the hospital’s computer network, and analysis was conducted using Microsoft Excel and Statistica software.

Patient Population

The study included both male and female patients aged 45-65 years old who were treated with the JAK-2 kinase inhibitors Tofacitinib, Baricitinib, or Upadacitinib. These patients had various rheumatologic conditions, including rheumatoid arthritis (RA), psoriatic arthritis (PsA), Crohn’s disease, and other rheumatologic diseases. Patients who were prescribed these medications but did not use them, as well as those under the age of 45 or above the age of 65, were excluded from the study.

Data Collection

Data were collected on the following variables: demographics including age, race, and sex; seropositivity status in the case of RA; cardiac history before the initiation of JAK inhibitor therapy; history of embolic events before and during the use of JAK-2 kinase inhibitors; history of diabetes mellitus (DM) and HbA1c levels; duration of JAK inhibitor treatment; dosage and type of JAK inhibitor (Table 1).

**Table 1:** Baseline characteristics of the study population TNFi, tumor necrosis factor inhibitor; JAK, janus kinase; DM, diabetes mellitus; RA, rheumatoid arthritis; PsA, psoriatic arthritis

Variable study population	N=72
Age, years	56.90 (45-66)
Race	
- White	72.22%
- Black	9.72%
- Hispanic	5.56%
- Indian	11.11%
- Asian	1.39%
Gender	
- Male	58.33%
- Female	41.67%
DM	
- No diabetes	83.33%
- DM	9.72%
- Prediabetes	6.94%
Previous TNFi use	38.89%
Mean duration	3.11 years
Diagnosis	
- RA	56.95%
- PsA	16.67%
- Alopecia	6.94%
- Colitis	19.44%
JAK treatment	
- Tofacitinib 11 mg	16.67%
- Tofacitinib 5 mg BID	25.00%
- Tofacitinib 10 mg BID	6.94%
- Upadicitinab 15 mg	29.17%
- Upadicitinab 30 mg	4.17%
- Baricitinib 2 mg	5.56%
- Baricitinib 4 mg	2.78%

Statistical Analysis

Data were analyzed using Microsoft Excel for initial data management and descriptive statistics. Statistica software was utilized for more advanced statistical analyses. Continuous variables were summarized using means and standard deviations, while categorical variables were summarized using frequencies and percentages.

Ethical Considerations

The study was approved by the Capital Health Institutional Review Board (IRB). All data were anonymized to protect patient confidentiality, and the study was conducted in accordance with the ethical standards of the institution.

## Results

**Figure 1:**
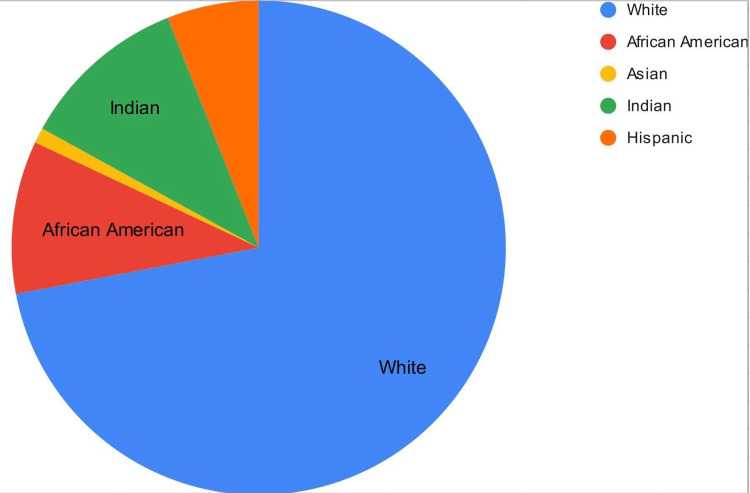
Patients' demographic

**Figure 2:**
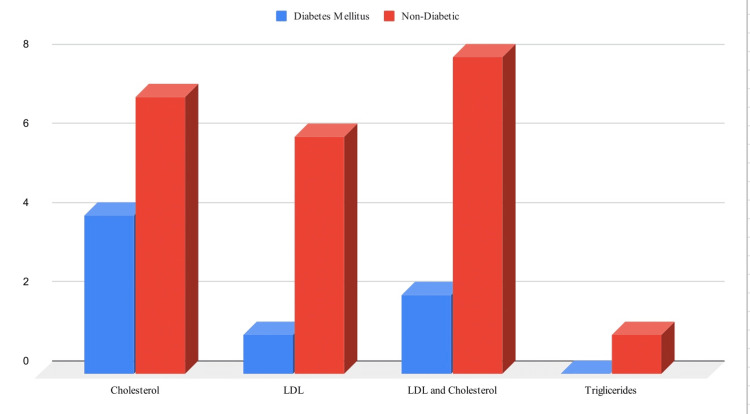
Lipid changes in correlation with DM DM, diabetes mellitus

Among 100 patients prescribed JAK inhibitors, 71 were included in the study (with an average treatment duration of 2.5 years). The majority of patients were white (72%), followed by Hispanic (6%), Indian (11%), African American (10%), and Asian (1%) ( Figure 1). There were no significant cases of VTE reported, with one patient developing pulmonary embolism (PE) during treatment with Tofacitinib while also having COVID-19, making it difficult to attribute it solely to the medication. Similarly, only one case of atrial fibrillation occurred. However, 43% (31 patients) experienced worsening of their lipid profile, with increased cholesterol (18%), LDL (12.5%), both LDL and cholesterol (11%) or triglycerides (1.5%). In subsequent visits, the lipid panel remained elevated. Patients primarily had RA (57%), followed by PsA (17%), colitis (20%), and alopecia areata (6%) (Table 2). With DM, 24 patients who experienced worsening of their lipid panel did not have a history of DM (Figure 2).

**Table 2:** Clinical features PsA, psoriatic arthritis; RA, rheumatoid arthritis

Clinical feature	Number of patients	Percentage (%)
RA	40	57
PsA	12	17
Colitis	14	20
Alopecia areata	5	6

## Discussion

JAK inhibitors are a class of medications that target JAK enzymes, which play a critical role in the signaling pathways of various cytokines and growth factors involved in processes such as immune function, cell growth, and hematopoiesis. By inhibiting JAKs, these drugs can modulate the immune system and are particularly useful in treating autoimmune diseases and certain cancers. JAK inhibitors work by blocking the activity of one or more of the JAK family members (JAK1, JAK2, JAK3, and TYK2), preventing the phosphorylation and activation of signal transducers and activators of transcription (STATs), which are downstream signaling molecules [5]. This disruption reduces inflammation and alters the immune response. JAK inhibitors are used in the treatment of several conditions, including RA, PsA, ulcerative colitis, myelofibrosis, polycythemia vera, and certain types of cancers such as myeloproliferative disorders [6].

The use of JAK inhibitors is increasing as more conditions are being treated with these medications. RA is one of the most common indications, affecting about 1% of the global population, and ulcerative colitis and other autoimmune diseases also represent significant portions of the patient population. Autoimmune diseases, which are commonly treated with JAK inhibitors, often have a higher prevalence in women; for instance, RA is approximately two to three times more common in women than in men, leading to a greater proportion of JAK inhibitor prescriptions being written for female patients [7].

Major cardiovascular events commonly manifest in various ways. Myocardial infarction (heart attack) may present with chest pain or discomfort, shortness of breath, nausea, vomiting, cold sweat, and lightheadedness. Stroke symptoms can include sudden numbness or weakness, particularly on one side of the body, confusion, trouble speaking, vision problems, difficulty walking, dizziness, loss of balance or coordination, and severe headache with no known cause. Heart failure often leads to shortness of breath, fatigue, swelling in the legs, ankles, and feet, rapid or irregular heartbeat, reduced ability to exercise, and persistent cough or wheezing [8].

VTE is a condition where blood clots form in the veins, encompassing deep vein thrombosis (DVT) and PE [9]. Risk factors for VTE include immobility, surgery (particularly orthopedic surgery), cancer and cancer treatment, hormone replacement therapy or oral contraceptives, pregnancy and the postpartum period, genetic clotting disorders, obesity, and smoking [6]. DVT symptoms include swelling in one or both legs, pain or tenderness in the leg, warmth in the affected area, and red or discolored skin. PE symptoms include shortness of breath, chest pain that may worsen when breathing in, rapid heart rate, and a cough that may produce bloody sputum. Diagnosis of VTE typically involves imaging studies such as ultrasound for DVT or CT pulmonary angiography for PE, with blood tests like D-dimer also aiding in diagnosis [7]. Treatment usually involves anticoagulant medications to prevent further clotting and allow the body to break down the existing clot, and in severe cases, thrombolytic therapy or surgical intervention might be necessary [10]. JAK inhibitors represent a significant advancement in the treatment of autoimmune diseases and certain cancers, with a notable impact on patient populations predominantly comprising women due to the higher prevalence of autoimmune conditions [11]. Cardiovascular events and VTE are critical considerations in patient management, requiring timely diagnosis and treatment to prevent serious complications [12].

## Conclusions

This retrospective study aimed to evaluate the usage patterns of JAK inhibitors in patients with rheumatologic conditions and assess their impact on cardiovascular outcomes.

Our analysis of 71 patients revealed no significant cases of VTE and only one instance of atrial fibrillation. However, nearly half of the patients experienced a worsening of their lipid profile. This suggests that while JAK inhibitors may be effective in controlling disease activity, potential cardiovascular risks need to be carefully considered when prescribing these medications.

Further research with larger patient populations and longer follow-up periods is necessary to fully understand the long-term cardiovascular safety profile of JAK inhibitors. Additionally, studies investigating the mechanisms underlying the impact of JAK inhibitors on lipid profiles could provide valuable insights for optimizing treatment strategies and minimizing adverse effects.
